# Parkinson's disease: Are gut microbes involved?

**DOI:** 10.1002/brb3.3130

**Published:** 2023-06-20

**Authors:** Abass Olawale Omotosho, Yusuf Amuda Tajudeen, Habeebullah Jayeola Oladipo, Sodiq Inaolaji Yusuff, Muritala AbdulKadir, Abdulbasit Opeyemi Muili, Oluwaseyi Muyiwa Egbewande, Rashidat Onyinoyi Yusuf, Zaccheaus Oluwaseun Faniran, Abdullateef Opeyemi Afolabi, Mona Said El‐Sherbini

**Affiliations:** ^1^ Department of Microbiology, Faculty of Pure and Applied Sciences Kwara State University, Malete‐Ilorin Ilorin Nigeria; ^2^ Department of Microbiology, Faculty of Life Sciences University of Ilorin Ilorin Nigeria; ^3^ Faculty of Pharmaceutical Sciences University of Ilorin Ilorin Nigeria; ^4^ Department of Epidemiology and Medical Statistics, Faculty of Public Health, College of Medicine University of Ibadan Ibadan Nigeria; ^5^ Department of Medicine, Faculty of Clinical Sciences Obafemi Awolowo University Ife Nigeria; ^6^ Department of Medicine Ladoke Akintola University of Technology Ogbomosho Nigeria; ^7^ Department of Microbiology, Faculty of Science University of Ibadan Ibadan Nigeria; ^8^ Faculty of Biomedical Sciences, Department of Microbiology and Immunology Kampala International University Bushenyi Uganda; ^9^ Narrative Medicine and Planetary Health, Integrated Program of Kasr Al-Ainy (IPKA), Faculty of Medicine Cairo University Cairo Egypt; ^10^ Invited Faculty the Nova Institute for Health Baltimore MD USA; ^11^ Department of Medical Parasitology, Faculty of Medicine Cairo University Cairo Egypt

**Keywords:** Braak's Theory, Gut‐brain axis, Gut Microbiome, Parkinson's Disease, Neurologial Disorders

## Abstract

**Introduction:**

Parkinson's disease (PD) is a neurodegenerative disorder that affects more than 10 million individuals worldwide. It is characterized by motor and sensory deficits. Research studies have increasingly demonstrated a correlation between Parkinson's disease and alternations in the composition of the gut microbiota in affected patients. Also, the significant role of prebiotics and probiotics in gastrointestinal and neurological conditions is imperative to understand their relation to Parkinson's disease.

**Method:**

To explore the scientific interaction of the gut‐microbiota‐brain axis and its association with Parkinson's disease, a comprehensive narrative review of the relevant literature was conducted. Articles were retrieved systematically from reputable sources, including PubMed, Science Direct, World Health Organization (WHO), and Advanced Google Scholar. Key search terms included are “Parkinson's Disease”, “Gut Microbiome”, “Braak's Theory”, “Neurological Disorders”, and “Gut‐brain axis”. Articles included in our review are published in English and they provide detailed information on the relationship between Parkinson's disease and gut microbiota

**Results:**

This review highlights the impact of gut microbiota composition and associated factors on the progression of Parkinson's disease. Evidence‐based studies highlighting the existing evidence of the relationship between Parkinson's disease and alteration in gut microbiota are discussed. Consequently, the potential mechanisms by which the gut microbiota may affect the composition of the gut microbiota were revealed, with a particular emphasis on the role of the gut‐brain axis in this interplay.

**Conclusion:**

Understanding the complex interplay between gut microbiota and Parkinson's disease is a potential implication for the development of novel therapeutics against Parkinson's disease. Following the existing relationship demonstrated by different evidence‐based studies on Parkinson's disease and gut microbiota, our review concludes by providing recommendations and suggestions for future research studies with a particular emphasis on the impact of the microbiota‐brain axis on Parkinson's disease.

## Graphical abstract

Parkinson is a neurodegenerative disease characterized by motor and non‐motor deficit, and affected an estimated 10 million people per year. Due to its association with gut microbiota via the gut microbiota–brain axis, this article discusses the role of gut microbiota composition and associated factors on Parkinson's disorder.

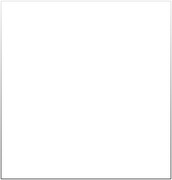

## 1 INTRODUCTION

Parkinson's disease (PD), also referred to as Parkinsonian disorder, represents the second most prevalent neurodegenerative condition following Alzheimer's disease. Primarily characterized by motor and sensory deficits (Bhattarai et al., 2021), Parkinson's disease affects approximately 10 million individuals worldwide, with nearly 1 million active cases in the United States alone, and an annual increase of nearly 60,000 new cases (Marras et al., 2018). PD is distinguished by the loss of dopaminergic neurons in the substantia nigra pars compacta (SNpc) and synucleinopathy, which entails the accumulation of insoluble polymers of α‐synuclein (αSyn) in neuronal bodies, leading to the formation of round lamellated eosinophilic cytoplasmic inclusions known as Lewy bodies (LB). These Lewy bodies contribute to neurodegeneration and neuronal death (Sulzer, 2007; Wolters & Braak, 2006). Cardinal motor features of Parkinson's disease include resting tremors, rigidity, slow movement, and shuffling gait (Kasper et al., 2014). Non‐motor symptoms (NMS) such as dementia, depression, and sensory and autonomic dysfunction often appear decades before the onset of motor symptoms (Heintz‐Buschart et al., 2018; Poewe, 2008). Most of these NMS go unnoticed during the prodromal stage, and treatment for PD is initiated only when motor symptoms appear; by which point more than 50% of the dopaminergic neurons in the substantia nigra may have degenerated (Cheng et al., 2010).

In individuals with PD, gastrointestinal symptoms, including compromised intestinal barrier function, frequently coincide with alternations in gut microbiota composition. Presently, researchers are captivated by the hypothesis that such a substantial population of microbes, possessing a vast genome, can significantly influence human behavior, physiology, and immunity. This along with the bidirectional communication known as the “gut microbiota–brain axis (GMBA)” is widely recognized and its dysregulation has been implicated in various diseases, emphasizing the need for a deeper understanding and novel therapeutic strategies (Nicholson et al., 2012). The influence of GM has been recognized in brain development and various neurological disorders such as Alzheimer's disease, multiple sclerosis, amyotrophic lateral sclerosis, anxiety, and stress, among others (Parashar & Udayabanu, 2017).

This review aims to provide a comprehensive understanding of Parkinson's disease (PD) in relation to the composition of the gut microbiota (GM) and its metabolites. Additionally, it explores the interplay between the GM, the blood–brain barrier (BBB), and the gut–brain axis. Various factors that contribute to alterations in GM observed, such as prebiotic, probiotic, and fecal microbiome transplantation (FMT) approaches, are examined for their potential in elucidating diagnostic biomarkers and modifying treatment strategies during the early stages of Parkinson's disease.

## 2 BRAAK'S THEORY AND PARKINSON'S DISEASE PATHOGENESIS

Prof. Heiko Braak, a prominent human pathologist, proposed a distinctive mechanism for the spread of αSyn pathology from the gut to the ventral brain through the vagus nerve (Braak et al., 2003). According to Braak's theory, αSyn pathology leads to the destruction of dopaminergic neurons (DA) in the SNpc, and the observed distribution pattern of Lewy body (LB) in postmortem human brains is attributed to this process (Braak et al., 2004). Synucleinopathy is characterized by cell death or loss of functional connectivity, ultimately resulting in neurodegeneration, dementia, and the development and progression of Parkinson's disease (Braak et al., 2003; Dodel et al., 2008). In the early stages of PD, LB pathology appears primarily in the olfactory bulb or the intermediate reticular zone (IZ) and the dorsal motor nucleus of the vagus nerve (DMV) in the medulla, with later progression affecting the midbrain disrupting SNc and other regions of the brain (Braak et al., 2004).

Symptoms of PD correlate with the presence of LB pathology in the substantia nigra pars compact, specifically within the dorsal motor nucleus of the vagal nerve and the olfactory bulb, which corresponds to stage 1 of the disease. In stage 2, motor symptoms become notable, accompanied by widespread LB deposits and neuritis in the medulla (Braak et al., 2004). Stage 3 is characterized by the progressive caudorostral and rostral–caudal spread of LB from the brain stem and olfactory areas, respectively, with αSyn positive deposits extending to the midbrain and forebrain before eventually reaching the SNpc. In stage 4, individuals exhibit PD symptoms along with apparent cell loss in the SNpc. Lastly, stages 5 and 6 are marked by the presence of cognitive dysfunction and severe motor symptoms (Braak et al., 2003; Dodel et al., 2008; Halliday & McCann, 2010).

The neuropathology of PD, based on observations by Braak et al. in autopsied patients, reveals a correlation between the amount and location of insoluble αSyn deposition, and the stage of the disease (Braak et al., 2002). The results of the neuropathology tests demonstrated a connection between the clinical symptoms and the Lewy pathology, suggesting a predictable progression of PD. Over time, there is an increase in the extent of Lewy pathology, with a progressive localization in the caudorostral direction (Braak et al., 2003; Dodel et al., 2008; Halliday & McCann, 2010). Based on the appearance and progression of LB and neuritis, Braak et al. proposed the “dual hit” theory, which is supported by numerous neuropathological and clinical observations. This theory suggests that unknown pathogens may enter either through the respiratory pathway or the gastric route, possibly through the nasal passage and swallowed saliva containing nasal secretion (Hawkes et al., 2009).

## 3 GASTROINTESTINAL TRACT SYMPTOMS IN PARKINSON'S DISEASE

About 40 years ago, despite early clinical reports and neuropathological findings providing clear evidence of gastrointestinal tract (GIT) involvement in PD, most neurologists and movement disorder specialists did not prioritize GIT symptoms in clinical practice (den Hartog Jager & Bethlem, 1960; Eadie & Tyrer, 1965; Warnecke et al., 2022). During that time, clinicians primarily focused on classical motor symptomatology. It was not until the early 1990s that clinical neurologists began to show increased interest in PD symptoms related to GIT due to the number of clinical studies and publications on the complex interaction of PD and GIT manifestations (Warnecke et al., 2022).

Parkinson's disease (PD) patients commonly experience a range of gastrointestinal symptoms, including constipation, drooling, dysphagia, abdominal pain, dyspepsia, and fecal incontinence (Pfeiffer, 2003; Salat‐Foix & Suchowersky, 2012). Symptoms such as abdominal pain, fecal incontinence, and disturbances of bowl movements are particularly prevalent when the effects of anti‐Parkinsonian medications begin to subside (Salat‐Foix & Suchowersky). However, approximately a quarter of patients on dopaminergic therapy may experience worsening nausea and constipation, which could be attributed to PD itself, its treatment, or a combination of both (Kulisevsky & Pagonabarraga, 2010; Salat‐Foix & Suchowersky, 2012). Population‐based studies have revealed that individuals with a long history of constipation have a 2.5‐to‐3‐fold increased risk of developing PD; recognizing constipation as a cardinal component in the premotor stage of PD (Abbott et al., 2003; Tolosa et al., 2007).

Alternation in gut microbiota has been observed in patients with prodromal or clinically established PD when compared with well‐controlled subjects ((Baizabal‐Carvallo, 2021; Zhu et al., 2022). The underlying pathology can be explained by increased intestinal permeability, abnormal aggregation of αSyn fibrils, aggravated neuroinflammation, oxidative stress, and decreased neurotransmitter production (Zhu et al., 2022). This along with decreased serum levels of LPS‐binding protein and altered blood microbiome in PD patients haveabnormal intestinal permeability, a condition known as ‘‘*leaky gut syndrome*’’ (Baizabal‐Carvallo, 2021). Evidence suggests that gut microbiota alternation ‘‘*dysbiosis’’* contributes to neuroinflammation and motor onset and progression of PD (Baizabal‐Carvallo, 2021; Qian et al., 2018) with recommendations for in‐depth investigations into the pathophysiology of PD and cutting‐edge therapeutic options (Baizabal‐Carvallo, 2021).

## 4 GUT MICROBIOTA ASSOCIATED WITH PARKINSON'S DISEASE

The gut microbiota (GM) is home to 100 trillion bacteria (as well as some fungi, archaea, and viruses), which is 10 times the number of cells in the human body (Franzosa et al., 2015). Furthermore, the collective genome of the gut microbiota contains approximately 3 million genes, which is 150 times more than the human genome (Franzosa et al., 2015). This human GM comprises a large ecological community with bacteria playing a major role, including Firmicutes, Bacteroidetes, Actinobacteria, Proteobacteria, Fusobacteria, and Verrucomicrobia (Rinninella et al., 2019). These bacteria contribute to the production of short‐chain fatty acids (SCFA), which possess anti‐inflammatory and antioxidant properties, and regulate the neuro–immuno–endocrine system while protecting the nervous system from neuronal damage (Bullich et al., 2021). Several studies have indicated the potential influence of gut microbiota (GM) on neurological functioning, including memory, learning, and cognition. Remarkably, one third of our GM is shared by most individuals, and the remaining two thirds are unique to each individual, much like a personalized identity card. The identification and characterization of the diverse array of gut microbes are still in their nascent stages, as an astounding 50% to 60% of these microbes have never been successfully cultured, possibly due to their adaptation for host‐to‐host transmission through spores (Browne et al., 2016; Walker et al., 2014).

The most common microbiota alteration associated with PD is the presence of *Helicobacter pylori* gastric infection*. H. pylori* infection has been found to induce the absorption of dopaminergic agonist, levodopa. Another microbiota alteration implicated in PD is small intestinal bacterial overgrowth (SIBO), which refers to the excessive growth of bacteria in the small intestine. Both *H. pylori* and SIBO have been shown to cause motor impairment and a reduction in the diversity level of GM in PD patients (Camci & Oguz, 2016; Fasano et al., 2013; Tan et al., 2014). A study by Hopfner et al. (2017) found a significant increase in the abundance of *Lactobacillaceae*, *Enterococcaceae*, and *Barnesiellaceae* in the PD patients compared to the control group, suggesting the potential role of GM in PD development (Hopfner et al., 2017). However, the study sample size was limited, and larger studies considering the effect of medications and further functional investigations of the gut microbiome are needed to elucidate the role of gut microbiota in PD pathogenesis.

In the intestinal mucosa of PD patients, there is an observed increase in bacteria belonging to the genus *Ralstonia* and a decrease in bacteria of the genus *Faecalibacterium*. Furthermore, PD subjects exhibited a significant decrease in the abundance of fecal bacteria belonging to the genus *Blautia, Coprococcus*, and *Roseburia* compared to individuals without PD (Forsyth et al., 2011). *Lactobacillus casei shirota*, a specific strain of gut microbiota, has been studied for its potential to improve bowel movement, alleviate constipation in PD and reduce PD morbidity (Cassani et al., 2011).

## 5 ALTERATION OF GUT MICROBIOTA AND ITS METABOLITES IN PARKINSON'S DISEASE

Alterations in the gut microbiota (GM), collectively referred to as gut dysbiosis, have been associated with various autoimmune, metabolic, and gastrointestinal diseases, including PD (Mukherjee et al., 2016; Tremlett et al., 2017). This is primarily due to the pro‐inflammatory state of the GIT, which results in an imbalance of bacteria that produce SCFA. Such pathology eventually leads to the formation and aggregation of αSyn (Shen et al., 2021). In patients with PD, alterations in the gut microbiota have been observed, with decreased abundance of Prevotellaceae, *Faecalibacterium*, and Lachnospiraceae and higher levels of *Lactobacillus* *, Clostridium*, Bifidobacteriaceae, Ruminococcaceae, Verrucomicrobiaceae, and Christensenellaceae (Shen et al., 2021).

Decreased abundance of Provotellaceae and increased abundance of Lactobacillicae have been associated with a reduced level of ghrelin hormone, which is involved in maintaining and protecting the function of nigrostriatal dopamine, a crucial neurotransmitter in PD pathogenesis (Andrews et al., 2009; Unger et al., 2011). Recently, a study investigated the relationship between microbiota, clinical symptoms, and PD staging (Scheperjans et al., 2015a, 2015b), found that decreased abundance of Provotellaceae was associated with gastrointestinal symptoms among PD patients (Mertsalmi et al., 2017). Gut microbiota alterations in terms of number and diversity, based on the Unified Parkinson Disease Rating Scale (UPDRS), significantly impact motor and NMS of PD and serve as reliable biomarkers of the disease (Hasegawa et al., 2015). Although different studies utilizing human and animal models have provided evidence to support the hypothesis of gut microbiota in the pathogenesis of PD, the scientific acceptance of this hypothesis is not yet fully conclusive. Research investigating the GM composition in PD patients using fecal samples has yielded inconsistent results, with variations in the specific gut microbiota responsible for dysbiosis across different studies.

## 6 FACTORS THAT CONTRIBUTE TO ALTERATIONS IN THE GUT MICROBIOTA IN PARKINSON'S DISEASE

Alterations in the GM in PD have been associated with several key factors, including host genetics, environmental toxins, dietary lifestyles, and aging. Research has shown that the composition of the gut microbiota can be influenced by host genetics, as certain microbiota taxa in the gut are heritable in humans (Goodrich et al., 2014). Lim et al. (2016) conducted a study to assess the impact of host genetics on the gut microbiota, beta diversity, and found that changes in the microbial community can be influenced by factors such as geography, nutrition, and ethnicity. While some bacteria, like Bfidobacteriaceae and Christensenellaceae, show consistent heritability, other bacteria exhibit variation in heritability due to the effects of these dynamic factors (Lim et al., 2016). Abnormalities in species host genes, such as nucleotide‐binding oligomerization domain 2 and fructosyltransferase 2, have also been associated with changes in the gut microbial population and increased susceptibility to diseases such as Parkinson's and Crohn's disease (Rausch et al., 2011; Rehman et al., 2011).

Environmental contaminants have been shown to affect the gut microbiota, leading to negative impacts on its composition and function (Tu et al., 2020). Various environmental factors, including metals, pesticides, antibiotics, and artificial sweeteners, have been implicated in these alternations (Tu et al., 2020). Antibiotics, for example, disrupt the gut microbiota by creating an unfavorable environment for commensal bacteria, resulting in a loss of diversity and an imbalance in composition (Lozupone et al., 2012). Additionally, antibiotics can alter the functional characteristics and overall makeup of the gut microbiome (Tu et al., 2020). The pesticide glyphosate has been found to inhibit beneficial gut bacteria, while the insecticide chlorpyrifos causes dysbiosis of the GM by increasing the number of *Bacteroides* spp. and reducing the concentration of *Lactobacillus* spp. and *Bifidobacterium* in the gut (Claus et al., 2016). The effects of aging and the physical environment on the gut microbiota are still not fully understood due to limited research in these areas (O'Toole & Jeffery, 2015). However, some insights have been gained from two examples of how the gut microbiota varies with age. First, age‐related dysbiosis, characterized by changes in the gut microbiota due to immune system deterioration and evasion of immune surveillance by some bacteria, has been observed. Second, the physiological tissue adaptability during host aging has been found to contribute to alterations in the gut microbiota (Bosco & Noti, 2021). Thus, alterations in gut microbiota in PD can be multifactorial and dynamically linked to environmental and genetic influences that merit further research to fully understand the mechanisms involved.

Growing research has been investigating the potential influences of confounding factors on PD pathogenesis (Safa et al., 2022; Yang et al., 2019). The notion is that lifestyle choices can impact PD pathophysiology and that smoking, and coffee consumption have been associated with a lower risk of PD; such effects are thought to be mediated by the GM (Scheperians et al., 2015). Studies have suggested that the positive effects of smoking and coffee may be linked to changes in the GM and reduction of intestinal inflammation (Biedermann et al., 2014). Furthermore, research has indicated that red wine and tea may have similar effects as coffee in reducing the risk of PD (Mills et al., 2015). However, in a PD mouse model, stress has been found to increase intestinal inflammation, gut permeability, endotoxemia, neuroinflammation, and dopamine loss, suggesting a potential role of stress in PD development (Dodiya et al., 2020).

Dietary factors such as intake of plant carbohydrates and fiber have been associated with higher levels of specific macronutrients that tend to be deficient in PD Patients (Arumugam et al., 6 ). The Western diet is characterized by high consumption of refined carbohydrates, hydrogenated oils, high‐calorie nutrient‐deficient meals, dairy products, and allergens, all of which may contribute to GM dysbiosis and the development of PD (Asherio et al., 2016; Fava et al., 2012).

Physical exercise has been shown to improve host metabolism and the gut environment, influencing GM and energy homeostasis (Monda et al., 2017). Furthermore, interactions between antibiotics and microbial toxins, such as lipopolysaccharide and epoxomicin, within the GM have the potential to cause significant alterations in GM composition, and inflammatory response and may impact neurological disorders, including PD (Parashay et al., 2017).

## 7 MICROBIOME, BLOOD–BRAIN BARRIER, AND GUT–BRAIN AXIS

The BBB, is a complex cellular barrier composed of various cell types, including capillary endothelial cells, tight junctions, basement membrane (fibrous matrix), neuroglial cells, glial podocytes (projection of astrocytes), and pericytes. The development of the BBB in the embryo occurs during the early fetal developmental stages. This complex cellular barrier protects the central nervous system (CNS) by restricting the permeability to various substances. Research conducted by Braniste et al. (2014) using germ‐free mice highlighted the significant influence of the gut microbiota on the development of BBB during early fetal stages. They found that germ‐free mice, which lacked normal intestinal microbiota, exhibited higher BBB permeability, and decreased expression of tight junction protein compared to mice with normal microbiota (Braniste et al., 2014). SCFA, such as butyrate, produced by microbial fermentation in the gut, is one of the neuroactive metabolites that can modulate the expression of tight junction proteins and affect the activity of the BBB's epigenetic histone deacetylase (Al‐Asmakh & Hedin, 2015; Mohajeri et al., 2018).

Bidirectional communication between the gut and the brain occurs through the vagal nerve, systemic metabolic pathways, and various neurological, endocrine, immunological, and humoral networks (Al‐Asmakh & Hedin, 2015; Mohajeri et al., 2018). The enteric nervous system (ENS), which surrounds the intestinal lumen, plays a crucial role in the interaction between the gut alimentation and the bacteria within, as it innervates the gastrointestinal system (Cosma‐Grigorov et al., 2020). It has been proposed that micro‐RNA “MiRNA” found in human fecal samples and extracellular vesicles can enter gut microbial cells, regulate gene expression, and subsequently shape the microbiome (Bienenstock et al., 2015). Experimental animal models using germ‐free mice with delayed stomach emptying and intestinal transit have demonstrated the influence of gut microbial colonization on intestinal sensory–motor function (Carabotti et al., 2015).

Numerous studies have provided evidence that GM can exert influence on the gut–brain axis through different mechanisms including endocrine, immunological, and direct neuronal processes. This body of research supports the hypothesis that the pathological degeneration associated with PD may originate from the gut and subsequently develop in the brain (Braak et al., 2006; Zhu et al., 2022). Additionally, preclinical studies have indicated that the vagus nerve might serve as a conduit for the transport of αSyn from the GIT to the brain (Cryan et al., 2020). Studies have consistently demonstrated that PD is frequently accompanied by gastrointestinal dysfunctions such as constipation, bloating, nausea, dysphagia, sialorrhea, vomiting, and gastroparesis (Zhu et al., 2022). Adams‐Carr et al. (2016) conducted a study revealing that individuals with constipation are at higher risk of developing PD compared to people without constipation, with constipation often preceding the clinical diagnosis of PD by more than 10 years.

Furthermore, GM can impact the synthesis of neurotransmitters, including gamma‐aminobutyric acid (GABA), and their metabolic by‐products, such as butyrate, which play a role in the nervous system function (Bienenstock et al., 2015). Bacterial fermentation products, particularly SCFA‐butyrate can regulate microglia, promote their development and activity in the CNS and maintain cerebral homeostasis (Erny et al., 2015). Additionally, bacterial SCFA‐butyrate controls serotonin production (Yano et al., 2015), which contributes to bowel peristalsis movement and overall GI motility (Sikander et al., 2009). Hence, the gut microbiota significantly contributes to the CNS homeostasis.

## 8 ANTI‐PARKINSONIAN TREATMENT

As there is currently no cure for PD, the primary goal of the therapy is to manage disease manifestations while minimizing adverse motor symptoms (Zahoor et al., 2018). Pharmacological management of PD can be categorized into symptomatic and neuroprotective (disease‐modifying) therapies. Although certain agents, such as MAO‐B inhibitors, creatine, and isradipine, have demonstrated promising neuroprotective effects, there is presently no proven therapy for disease modification (Hauser et al., 2020). Additionally, deep brain stimulation which is the preferred surgical procedure for PD, or neuro ablative lesion surgeries are among the currently available surgical treatment options.

## 9 PHARMACOLOGICAL THERAPY FOR PARKINSON'S DISEASE

### 9.1 Symptomatic therapy

PD symptoms are categorized into the motor, the predominant features of the disease, and NMS. The motor disorder primarily arises from the selective depletion of dopamine‐producing neurons in the SNpc. Thus, the cornerstone of PD treatment involves dopaminergic drugs designed to mimic dopamine action in the depleted striatum (Jankovic, 2008; Sveinbjornsdottir, 2016). There are three main classes of drugs to manage motor symptoms of PD, including levodopa, monoamine oxidase (MAO)‐B inhibitors, and dopamine agonists. Other agents, such as anticholinergic and antiviral agents (amantadine), have shown beneficial effects, particularly as second‐line treatment options; however, their efficacy is limited and they may induce neuropsychiatric side effects, such as confusion and hallucination (Jaquet et al., 2009). It is common practice in the pharmacological treatment of PD to initiate therapy with a low dose and adjust it based on the patient's response to treatment, primarily to minimize the adverse effects of anti‐PD drugs.

### 9.2 Levodopa

In the treatment of Parkinson's disease (PD), levodopa, in combination with carbidopa, a peripheral dopa decarboxylase inhibitor, is considered the mainstay of symptomatic management (Jaquet et al., 2009). Levodopa undergoes conversion into dopamine upon crossing the BBB through the action ofdopa decarboxylase. Carbidopa is administered alongside levodopa to prevent this conversion in the peripheral system, as dopamine produced peripherally cannot penetrate the BBB. The therapeutic effects of levodopa are characterized by rapid and substantial symptomatic relief, which often lasts for several hours, particularly during the early phase of the disease (Mayo Clinic, 2022). However, as PD progresses, the duration of levodopa's effectiveness frequently diminishes, necessitating more frequent dosing (Jaquet et al., 2009). Prolonged use of levodopa is also associated with significant adverse effects, particularly motor fluctuations, and dyskinesias, which are often difficult to manage (Zahoor et al., 2018). Consequently, physicians often opt to delay the initiation of levodopa therapy if other viable alternatives effectively address the symptoms of PD (The National Collaborating Centre for Chronic Conditions, 2006).

### 9.3 Dopamine agonists

Dopamine agonists, including Ropinirole and Pramipexole, exert their therapeutic effect by directly binding to dopaminergic receptors, bypassing the need to convert to dopamine. These agents provide moderate symptomatic relief and have been found to delay the onset of dyskinesia when compared to levodopa. Consequently, they are often preferred as initial therapy, particularly in younger patients (Zahoor et al., 2018). Nevertheless, research studies have also highlighted that the use of dopamine agonists is associated with approximately a 15% increase in adverse events, notably impulse control disorders, which have adverse social consequences (Zahoor et al., 2018). Additionally, although imaging studies have suggested that these agents may possess antioxidant effects and potentially reduce the loss of dopaminergic neurons, there is currently no evidence supporting their ability to modify the progression of the disease (Ahlskog, 2003).

### 9.4 Monoamine oxidase‐B inhibitors

MAO‐B inhibitors, such as selegiline, rasagiline, and safinamide, act by inhibiting MAO, an enzyme responsible for the breakdown of dopamine in the brain. By blocking this enzyme, (MAO)‐B inhibitors increase dopaminergic activity through the preservation of endogenous dopamine (Ahlskog, 2003). These agents are commonly considered in the early stage of Parkinson's disease as they provide mild symptomatic benefits and adverse effects, with gastrointestinal effects being the most frequently reported complaint (Zahoor et al., 2018). A systematic review conducted by Cochrane indicated that (MAO)‐B inhibitors improve quality of life indicators by approximately 25% (Caslake et al., 2009). Furthermore, these agents, particularly selegiline, have demonstrated some neuroprotective effects in animal studies by safeguarding dopamine cells in mice from 1‐methyl‐4‐phenyl‐1,2,3,6‐tetrahydropyridine (MPTP) toxicity, an effect not solely attributable to MAO‐B blockade (Tatton & Greenwood, 1991).

### 9.5 Other classes of anti‐PD drugs

Anti‐Parkinson's disease agents with distinct mechanisms of action include catechol‐O‐methyl transferase (COMT) inhibitors, such as entacapone and opicapone. These inhibitors function by blocking the peripheral metabolism of levodopa to 3‐O‐methyldopa by COMT. Consequently, they increase the half‐life of levodopa, allowing for more substantial amounts of the medicine to cross the BBB and prolong its therapeutic effects (Tatton & Greenwood, 1991). COMT inhibitors are commonly used as an adjunct to levodopa, particularly in the advanced stage of PD, to reduce the off‐time period (Zahoor et al., 2018). Another class of anti‐PD drugs is anticholinergics, which works by potentially restoring and maintaining a normal balance between dopamine and acetylcholine in individuals with PD (Goldenberg, 2008). The reduction of dopaminergic neurons in PD disrupts the typical dynamics between dopamine and acetylcholine in the brain (Goldenberg, 2008). While anticholinergic drugs provide tremor relief in about 50% of patients, their efficacy in treating bradykinesia and rigidity is limited. Additionally, they are associated with neuropsychiatric and parasympathetic blockade side effects (Zahoor et al., 2018). Amantadine, an antiviral agent, has also exhibited anti‐parkinsonian effects, although its precise mechanism of action remains unclear. It is hypothesized that amantadine enhances CNS dopaminergic responses by stimulating dopamine release from the storage sites and blocking its reuptake (Goldenberg, 2008). Due to its characteristics, amantadine is typically used to reduce the severity of levodopa‐induced dyskinesias (Sawada et al., 2010). While generally well‐tolerated, potential side effects include nausea, headache, hallucinations, confusion, livedo reticularis, and others (Parkinson's Disease Toolkit, 2022). Emerging novel approaches, such as gene therapy, are also being explored for PD treatment. Gene therapy involves utilizing gene transfer techniques to enhance the availability of dopamine in the striatum and reduce the actions of the subthalamic nucleus through local stimulation of GABA expression (Witt & Marks, 2011). However, these approaches are still in the developmental stage, and further studies are necessary to ascertain their effectiveness.

### 9.6 Microbiota as a therapeutic target in PD (microbes associated with PD drug metabolism)

The complexity of Parkinson's disease pathophysiology and the absence of disease‐modifying therapies have stimulated the exploration of new therapeutic agents (Zhu et al., 2022). Current treatments primarily focus on symptom relief, such as dopamine replacement therapy with levodopa (L‐dopa), which is associated with various side effects (Zhu et al., 2022). Consequently, there is a growing demand for curative therapies that extend beyond symptom alleviation (Zhu et al., 2022). Interventions targeting the gut microbiota, such as fecal microbiota transplantation (FMT) have gained attention as potential treatments for PD. FMT involves transferring fecal matter from a healthy donor into the GIT of a patient, aiming to restore a healthy bacterial composition and undue gut dysbiosis (Fan et al., 2022). This process holds promise to enhance the selectivity of PD treatments (Fan et al., 2022). Additionally, the use of prebiotics (nutrients metabolized by probiotic bacteria to stimulate bacterial growth), and synbiotics (a combination of probiotics and beneficial bacteria) has been suggested to restore a healthy microbiota and offer therapeutic benefits (Gagliardi et al., 2018).

Recent advances in understanding the gut–brain axis interplay in PD have revealed the close interaction between the gut microbiota and PD, particularly in the early stages (Zhu et al., 2022). Consequently, gut microbiota emerges as a promising diagnostic tool and therapeutic target for PD (Zhu et al., 2022). Various therapeutic options, including probiotics, psychobiotics, prebiotics, synbiotics, postbiotics, FMT, dietary modifications, and traditional Chinese medicines, are being explored to modulate the gut microbiota composition and the microbiota–gut–brain axis. These approaches represent a forward‐looking strategy for PD treatment, aimed at influencing the initial step in the neurodegeneration cascade (Zhu et al., 2022). However, further research is warranted to fully comprehend the efficacy, interactions, and long‐term therapeutic approaches.

In the context of medication prescribed for neurological conditions, interactions between intestinal symptoms, gut bacteria, and drug metabolism have been observed. Drug fate and activity are influenced not only by the host but also by microorganisms present in the gut (Cryan et al., 2020). L‐dopa, the primary prescription for PD treatment, may be affected by the gut microbiota, potentially impacting its availability, efficacy, and side effects (Rekdal et al., 2019). For example, *Enterococcus faecalis* has a conserved tyrosine decarboxylase enzyme capable of metabolizing L‐dopa, potentially impacting its effectiveness through premature conversion to dopamine (Rekdal et al., 2019; Zhu et al., 2022). Furthermore, *Eggerthella lenta* has been reported to dehydroxylate dopamine using a molybdenum‐dependent enzyme. Both *E. faecalis* and *E. lenta* metabolize L‐dopa in the human gut microbiota, and the inhibition of gut microbial L‐dopa metabolism has been observed with (S)‐α‐fluoromethyltyrosine (AFMT) (Rekdal et al., 2019).

### 9.7 Prebiotic, probiotic, and fecal microbiome transplantation approach in PD therapeutics

Restoring eubiosis and homeostasis in the gut of patients can be achieved through the administration of mixtures containing prebiotics and probiotics. Prebiotics are dietary ingredients that are resistant to digestion by the host and induce changes in the composition or activity of the gastrointestinal flora (Brown & Goldman, 2020; Cantu‐Jungles et al., 2019; Gibson et al., 2010; Van Laar et al., 2019). In contrast, probiotics are living microbes, that are isolated and often formulated into capsules, tablets, or powders or administered in supplements, yoghurts, or other fermented foods for health purposes (Brown & Goldman, 2020; Hill et al., 2014; Van Laar et al., 2019). Common strains of probiotics include *Lactobacillus* and *Bifidobacterium*; which have been extensively studied for their health benefits. In the context of PD, the administration of *Lactobacillus* and *Bifidobacterium* throughout 1 to 3 months has demonstrated ongoing effectiveness in treating constipation (Hill et al., 2014). Both prebiotics and probiotics mixtures have shown significant clinical benefits in gastrointestinal diseases, such as maintaining remission in inflammatory bowel disease, reducing the recurrence of *Clostridium difficile* infection, and preventing antibiotic‐associated diarrhea (Brown & Goldman, 2020; Varankovich et al., 2015). In a trial conducted by Barichella and colleagues in 2016, 120 PD patients were randomly selected to consume either fermented milk containing prebiotic fiber and various probiotic strains or a placebo once daily for 4 weeks (Barichella et al., 2016). After 2 weeks, the group consuming fermented milk experienced a greater number of complete intestinal movements and a larger decrease in the use of other laxatives than the patients who consumed a placebo (Barichella et al., 2016). The presence of dietary fiber in the intervention likely contributed to increased stool frequency, thereby improving constipation (Brown & Goldman, 2020; Yang et al., 2012). It remains uncertain whether probiotics have the same impact on constipation in PD patients due to potentially different underlying causes of constipation in this population (Brown & Goldman, 2020; Dimidi et al., 2014). However, the beneficial effects of probiotics may involve a direct modulation of gut motility, mucin secretion, or immunomodulatory interactions (Dimidi et al., 2017).

Another approach to restoring eubiosis in the gut is FMT, also known as microbial replacement therapy. FTM involves transferring liquid fecal material from a healthy donor to a patient with dysbiosis through various methods such as orally administered capsules, gastroduodenal endoscopy, nasoduodenal tube, colonoscopy, or enema (Brown & Goldman, 2020; Van Laar et al., 2019). The effectiveness of FMT relies on the donor's ability to provide the required taxa capable of restoring metabolic deficits in the recipient patient and donor–recipient compatibility, which will be influenced by genetic factors such as differences in innate immune responses or environmental factors such as diet, xenobiotic exposure, and microbial interactions (Van Laar et al., 2019; Wilson et al., 2019).

Compared to probiotics, FMT offers advantages in terms of providing a more complex, complete, and stable assortment of intestinal microorganisms that can restore and maintain eubiosis (Van Laar et al., 2019). However, probiotics can be administered for longer periods allowing for a more targeted and enduring effect. In contrast, FMT primarily renews the luminal microbiome and may not induce significant changes in the mucosal microbiome if the transplanted stool is washed out before fostering eubiosis or a deeper change in the mucosal microbiome (Van Laar et al., 2019).

Furthermore, pathogenesis in PD may be facilitated by changes in the gut microbiota and related chemicals. According to some research, probiotics can reduce the effects of Parkinson's disease (PD) by improving mitochondrial dynamics and homeostasis (Safa et al., 2022). However, randomized clinical trials to examine the efficiency of microbial products, probiotic‐based supplements, and dietary intervention in reversing gut microbial dysbiosis in Parkinson's disease remain a huge unmet need (Safa et al., 2022). Additionally, based on different eating habits, constipation, drugs, intestinal microflora, and living environment, it is difficult to assess whether a single probiotic has the same therapeutic effect on patients in different areas at the moment (Yang et al., 2021). More importantly, there are numerous strains of probiotics, and individual strains of different probiotics may have varied effects, necessitating additional research to assess the efficacy of various strains (Yang et al., 2021). Table 1 summarized studies of randomized, placebo‐controlled clinical trials on pro/prebiotics in PD.

**TABLE 1:** Summarized studies of randomized, placebo‐controlled clinical trials on the role of pro/prebiotics in Parkinson's disease.

S/N	Study samples	Symptoms	Therapeutic interventions	Findings	Study limitations and discussion	References
1.	A preliminary trial enrolled 15 individuals with PD who underwent FMT treatment, 11 men and 4 women. On average, the age was 61 years old.	The patient's circumstances were thoroughly evaluated, including disease duration, Hoehn–Yahr classification, and therapeutic medicines.	Patients' non‐relatives who were healthy donors between the ages of 18 and 24 were chosen. Donors’ fecal microbiota were isolated and purified. Less than an hour passed between the emission of feces and the transplantation of the freshly purified fecal microbiota suspension into the patient's intestines. FMT was carried out through the gastrointestinal and colonic routes.	Significant improvement in sleep quality and reduction of motor symptoms, anxiety, and depression in 10 PD patients who got FMT via colonoscopy but not by gastrointestinal tube.	The absence of a placebo as a randomized control and the use of self‐control to examine before and after FMT treatment. There were just five instances that received nasoin‐testinal FMT. A brief (3‐month) follow‐up period and there was no analysis of the composition of gut microbiota.	Xue et al. (2020)
2.	A single‐center, double‐blind, randomized, placebo‐controlled experiment. A total of 72 patients were enrolled in the study.	Eligible patients were 40 years and above and had a PD diagnosis determined by neurological specialists using the Queen Square Brain Bank Criteria, with symptoms lasting at least 6 months. The subjects met the Rome IV criteria for functional constipation at least once per week for the previous 3 months.	For 4 weeks, the participants were given either multi‐strain probiotic capsules (*N* = 34) or a placebo pill with the same appearance (*N* = 38).	Measures of constipation‐related quality of life (such as the number of SBM per week, stool consistency, and satisfaction with the intervention) were considerably improved by a multi‐strain probiotics treatment.	Levodopa pharmacokinetics alterations are not measured. An extremely brief intervention period. To discover the most effective strains, dosages, and treatment duration as well as to assess efficacy and safety over the long term, additional research is required.	Tan et al. (2014)
3.	Randomized controlled studies on men and women between the ages of 50 and 80, patients with PD‐related constipation.	Men and women between the ages of 50 and 80 with an idiopathic Parkinson's disease diagnosis, Hoehn–Yahr (H–Y) stage I–III, the ability to walk unassisted for the necessary gait tasks, a stable dose of anti‐Parkinsonian medication for at least 2 weeks before the study's start, the ability to follow simple instructions, the absence of uncontrolled chronic diseases, and fulfillment of Rome I–IV criteria for functional constipation.	Probiotics were tested in randomized controlled studies	To improve the consistency of stools and treatment satisfaction.	Probiotics have been shown by this study to improve quality of life, decrease the incidence of adverse events, and ease constipation. A meta‐analysis of the UPDRSIII scale will be carried out once there is sufficient research to do so to better understand how probiotics enhance non‐motor PD symptoms.	Yang et al. (2021)
4.	Between 2013 and 2014, a random sample of 40 PD patients (17 males, 23 females; mean age 76.05 years) were treated with levodopa or dopamine agonists.	Constipation with an unpleasant sensation of incomplete feces, abdominal discomfort, and bloating.	Probiotic supplementation (60 mg, a blend of two lactic bacteria: *Lactobacillus acidophilus* and *Bifidobacterium infantis*), twice a day, 1 hour after meals for 3 months. Trimebutine was utilized as a positive control and comparative group (200 mg/day, 3/day, *N* = 20/group).	Probiotic supplementation reduced bloating and stomach pain in PD patients, similar to trimebutine therapy.	Only participants with mild to moderate GI‐NMS were included in the study. A small group of patients with Parkinson's disease and lower GI‐NMS were studied. There is no obvious relationship between the duration and severity of Parkinson's disease and the severity of constipation. Other related autonomic abnormalities, such as nocturia, orthostatic hypotension, and excessive sweating, were present in nearly half of the patients.	Georgescu et al. (2016)
5.	11 PD patients with constipation symptoms participated in a prospective, single study.	One of the individuals experienced diarrhea, fever, or other uncomfortable symptoms. The Hoehn–Yahr (H–Y) Grade, the Unified Parkinson's Disease Rating Scale (UPDRS) II Score, the non‐motor symptom scale (NMSS), the PAC‐QOL score, the Wexner constipation score, and the body mass index (BMI) (mm/kg^2^) are among the baseline parameters. Patients with PD had an average illness duration of 7.18 to 3.25 years.	Approximately 40 to 50 mL of frozen fecal microbiota was suspended, fresh every time, and transplanted into the colon within 2–4 min of suspension via a nasoduodenal tube.	The effects of Fecal Microbial Transplant on the gut microbiota and constipation symptoms were improved in all patients.	To guarantee the effectiveness and safety of FMT in the treatment of PD, a future study will need to be conducted with a comparatively smaller sample size and a bigger group.	Kulisevsky et al. (2010)

## 10 LIMITATIONS IN THE ROLES OF GUT MICROBIOTA IN PARKINSON'S DISEASE

While numerous scientific studies have provided evidence supporting the role of the gut microbiota in PD, there are significant limitations to consider. The first limitation is that data and results from human metagenomics studies lay the groundwork for future research, generating concrete hypotheses that can be experimentally tested to understand the various roles the gut microbiota may play in PD (Weiner et al., 2023). Additionally, some studies have suggested that confounding variables and reverse causality could explain the observed relationship between gut microbiota and PD. Factors such as PD‐related medication usage (e.g., Levodopa), advanced disease stages in the majority of cases, and differences between PD patients and controls in terms of age, gender, and ethnicity may impact the results. Furthermore, the heterogenicity in available metadata makes it difficult to account for detailed confounding factors like co‐morbidities, drug therapies, and diet. As a result, the causal link between microbiota alterations and PD cannot be confirmed based on currently available data. Future research should aim to standardize recruitment criteria and focus on early‐stage drug‐naive individuals to assess the causation between microbiome alterations and PD.

In addition to the limitations mentioned above, studies investigating interactions between specific genetic variants in the synuclein alpha (SNCA) region, and the gut microbiota PD require larger sample sizes to achieve sufficient statistical interpretations (Wallen et al., 2021). Interaction studies often have lower significance levels compared to association studies, and in this context, the study of opportunistic pathogens and their relationship with PD was limited by sample size (Wallen et al., 2021).

Similarly, the study by Wallen et al. in 2020 also faced limitations in sample size and resolution. To determine if PD is associated with polymicrobial clusters, larger sample sizes, and shotgun metagenomic sequencing are needed. The use of 16S amplicon sequencing in their study did not provide resolution at the species and gene level, and no efforts were made to detect viruses and fungi in the polymicrobial cluster (Wallen et al., 2020).

## 11 RECOMMENDATIONS AND CONCLUSION

Growing evidence strongly suggests a significant association between the gut microbiome and Parkinson's disease (PD). The impact of gut health on PD pathophysiology is now recognized to be more substantial than previously believed, with Braak's hypothesis providing a promising framework for understanding the influence of the GIT status. This hypothesis is supported by numerous pathological studies investigating the role of αSyn and other neuropathological changes in the progression of the disease. Clinical studies have also contributed to the growing body of evidence by recognizing GIT symptoms in the early stages of PD may serve as important indicators for earlier diagnosis and improved overall management. However, it should be acknowledged that the GM may not be universally implicated in the prodromal phase of all PD patients, thus, necessitating a focused systemic approach as recommended by Heinzel et al. (2018). Additionally, limited research has been conducted on the role of gut symptoms in the early stages of PD. Identifying and monitoring GIT symptoms in the early phases may potentially enable the correlation of these symptoms with the initial pathophysiological changes.

The significant involvement of the gut microbiota in Parkinson's disease provides a promising avenue for investigating how specific bacterial strains and metabolites may regulate CNS processes, thereby contributing to motor dysfunction. Conducting prospective cohort studies on PD patients to investigate the efficacy of specific probiotics would be a valuable step toward determining the effectiveness of such treatments. Furthermore, studying the effects of different diets on gut flora would be beneficial in examining the hereditary component and familial causes of PD. This approach has been shown to reduce variability in background dietary patterns and microbiota composition among individuals. Moreover, human research exploring the relationship between microbiota and PD in various research is still in its infancy. Therefore, conducting longitudinal studies encompassing both the prodromal and clinical phases while considering multi‐omics approaches, environmental factors, and detailed phenotypic clinical data, would significantly enhance our understanding of the connections between the microbiota and Parkinson's disease.

## CONFLICT OF INTEREST STATEMENT

The authors declare no conflicts of interest.

## FUNDING

Authors have received no funding

### PEER REVIEW

The peer review history for this article is available at https://publons.com/publon/10.1002/brb3.3130

## Data Availability

None
